# Supplementary figures and images for: Evaluating probabilistic programming and fast variational Bayesian inference in phylogenetics
Source: PeerJ. 2019 Dec 18;7:e8272. doi: 10.7717/peerj.8272 (PMC6966998; doi:10.7717/peerj.8272)

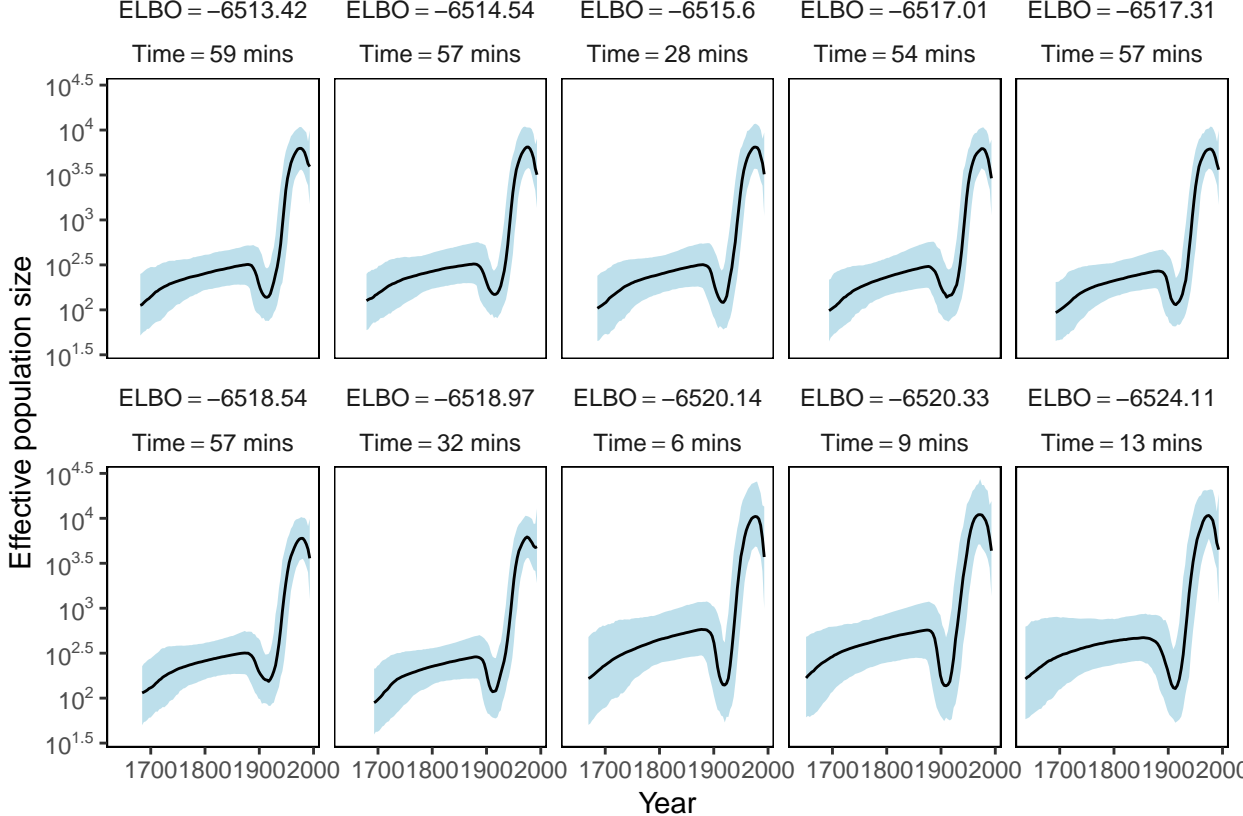

Supplement: Supplemental Information 1 — 10 Independent replicates are shown. [file peerj-07-8272-s001.pdf]

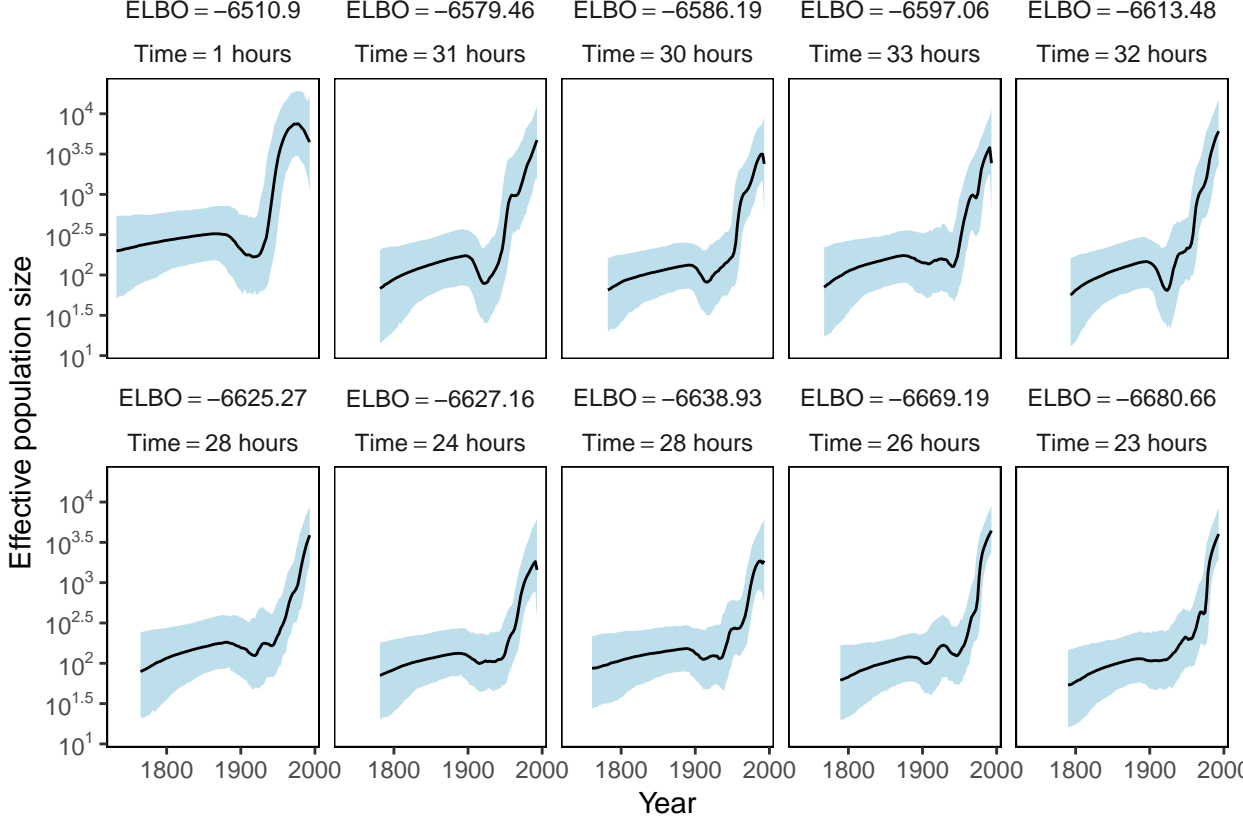

Supplement: Supplemental Information 2 — 10 Independent replicates are shown. [file peerj-07-8272-s002.pdf]
